# Multimodal therapy and use of adjunctive therapies to BoNT-A in spasticity management: defining terminology to help enhance spasticity treatment

**DOI:** 10.3389/fneur.2024.1432330

**Published:** 2024-08-30

**Authors:** Rajiv Reebye, Luis Jorge Jacinto, Alexander Balbert, Bo Biering-Sørensen, Stefano Carda, Nathalie Draulans, Franco Molteni, Michael W. O’Dell, Alessandro Picelli, Andrea Santamato, Monica Verduzco-Gutierrez, Heather Walker, Joerg Wissel, Gerard E. Francisco

**Affiliations:** ^1^Division of Physical Medicine and Rehabilitation, Faculty of Medicine, University of British Columbia, Vancouver, BC, Canada; ^2^Adult Rehabilitation Service, Alcoitão Rehabilitation Medicine Center, Estoril, Portugal; ^3^Department of Adaptive Physical Training, Ural University of Physical Education, Sverdlovsk Regional Hospital for War Veterans, Yekaterinburg, Russia; ^4^Neurological Department, Copenhagen University Hospital Rigshospitalet, Copenhagen, Denmark; ^5^Neuropsychology and Neurorehabilitation, Lausanne University Hospital, Lausanne, Switzerland; ^6^Department of Rehabilitation, Libra Rehabilitation and Audiology, Eindhoven, Netherlands; ^7^Villa Beretta Rehabilitation Center, Valduce Hospital, Costa Masnaga, Italy; ^8^Clinical Rehabilitation Medicine, Weill Cornell Medicine and Neuro Rehabilitation Consultants, New York, NY, United States; ^9^Neuromotor and Cognitive Research Center, Section of Physical and Rehabilitation Medicine, Department of Neurosciences, Biomedicine and Movement Sciences, University of Verona, Verona, Italy; ^10^Unit of Spasticity and Movement Disorders, Division of Physical Medicine and Rehabilitation, University Hospital of Foggia, Foggia, Italy; ^11^Department of Rehabilitation Medicine, University of Texas Health Science Center at San Antonio, San Antonio, TX, United States; ^12^Physical Medicine and Rehabilitation, Ralph H. Johnson VA Medical Center, Charleston, SC, United States; ^13^Neurology and Psychosomatic at Wittenbergplatz, Berlin and University Potsdam, Potsdam, Germany; ^14^Department of Physical Medicine and Rehabilitation, The University of Texas Health Science Center McGovern Medical School, Houston, TX, United States; ^15^Physical Medicine and Rehabilitation, The Institute for Rehabilitation and Research (TIRR) Memorial Hermann Hospital, Houston, TX, United States

**Keywords:** augmentation, combined modality, muscle spasticity, muscular paresis, botulinum neurotoxin, recovery of function

## Abstract

Spasticity management should be provided within the context of a comprehensive person-centered rehabilitation program. Furthermore, active goal setting for specific spasticity interventions is also important, with a well-established “more is better” approach. It is critical to consider adjunctive therapy and multimodal approaches if patients are not attaining their treatment goals. Often used interchangeably, there may be confusion between the terms adjunctive and multimodal therapy. Yet it is imperative to understand the differences between these approaches to achieve treatment goals in spasticity management. Addition of a secondary pharmacologic or non-pharmacologic treatment to optimize the efficacy of the initial modality, such as adding electrical stimulation or casting to BoNT-A, is considered an adjunctive therapy. Adjunctive therapy is time-specific and requires the added therapy be initiated within a specific period to enhance the primary treatment; usually within 2 weeks. Multimodal therapy is an integrated, patient-centric program of pharmacologic and non-pharmacologic strategies utilized in a concurrent/integrated or sequential manner to enhance the overall treatment effect across a variety of spasticity-associated impairments (e.g., neural and non-neural components). Moreover, within a multimodal approach, adjunctive therapy can be used to help enhance the treatment effect of one specific modality. The objectives of this paper are to clarify the differences between adjunctive and multimodal therapies, provide a brief evidence-based review of such approaches, and highlight clinical insights on selecting multimodal and adjunctive therapies in spasticity management.

## Introduction

The management of spasticity should be provided within the context of a comprehensive patient-specific, goal-centered, team-based rehabilitation program and include considerations for utilizing both non-pharmacological and pharmacological interventions (1, 2). Focal non-pharmacological interventions include modalities such as electrical stimulation, extracorporeal shockwave therapy (EC-SWT), stretching, splinting and casting, while generalized non-pharmacological interventions can often be implemented by Occupational/Physiotherapy such as posture or physical management, transfer, or dynamic movement. These interventions can be combined (3–5) within a multimodal therapy program (6) to achieve treatment goals.

Botulinum toxin type-A (BoNT-A) has utility for treating focal, multifocal, and segmental spasticity by decreasing muscle overactivity and the rate of development of contracture formation. If treatment goals, no matter if active or passive, are not considered attainable with BoNT-A alone and especially if not achieved after treatment with BoNT-A alone, consideration of adjunctive therapy and multimodal strategies is important. To choose the appropriate interventions for adjunctive or multimodal therapy, it is critical to better understand the definition of “adjunctive” and “multimodal.” In addition, it is imperative to categorize the underlying neural and non-neural factors of spasticity when considering incorporating adjunctive or multimodal treatment approaches.

During the acute phase of illness, central nervous system (CNS) damage may lead to paralysis and immobilization of the tendon and muscle in the shortened position, leading to soft tissue plastic rearrangements and contracture. The delayed phase involves CNS plastic rearrangements, including supraspinal and spinal rearrangements leading to muscle overactivity (7).

Therapeutic approaches in focal spasticity management to maximize tendon or muscle stretching can improve alignment and reduce contracture formation. Combination therapies such as those including, for example, stretching, splinting, and BoNT-A are considered multimodal. There is still no consensus on the minimum or maximal number of multimodal therapies necessary to enhance spasticity treatment outcome(s) (8). The addition of a secondary treatment to optimize the efficacy of, for example, BoNT-A injection(s) such as electrical stimulation or casting, is considered as an adjunctive therapy (4, 5, 9). Unlike a multimodal approach, adjunctive therapy is time-specific and requires the added therapy to be initiated within a specific period to enhance the primary treatment; usually within 2 weeks (4, 5).

The concept of a multimodal approach was described by Esquenazi and colleagues (2010) as part of an international consensus statement (6). Yet there is confusion between the terms adjunctive and multimodal therapy as they are often used interchangeably. Understanding the terminology of both multimodal and adjunctive therapy is important to help patient spasticity management, as well as when designing future spasticity research studies in multimodal and adjunctive therapies.

The objectives of this paper are to clarify the differences between adjunctive and multimodal therapies for non-pharmacological conservative spasticity management, provide a brief evidence-based review of such approaches, and highlight clinical insights and pearls on how to choose multimodal therapies in spasticity management and adjunctive therapy to help optimize BoNT-A effects.

Understanding the contrast between adjunctive and multimodal therapies can help improve research methodology, optimize treatment goals (both passive and active), reduce the quantity of BoNT-A injections, and potentially prolong the effect of BoNT-A when considering adjunctive therapy.

## Defining treatment approaches

**Figure 1:**
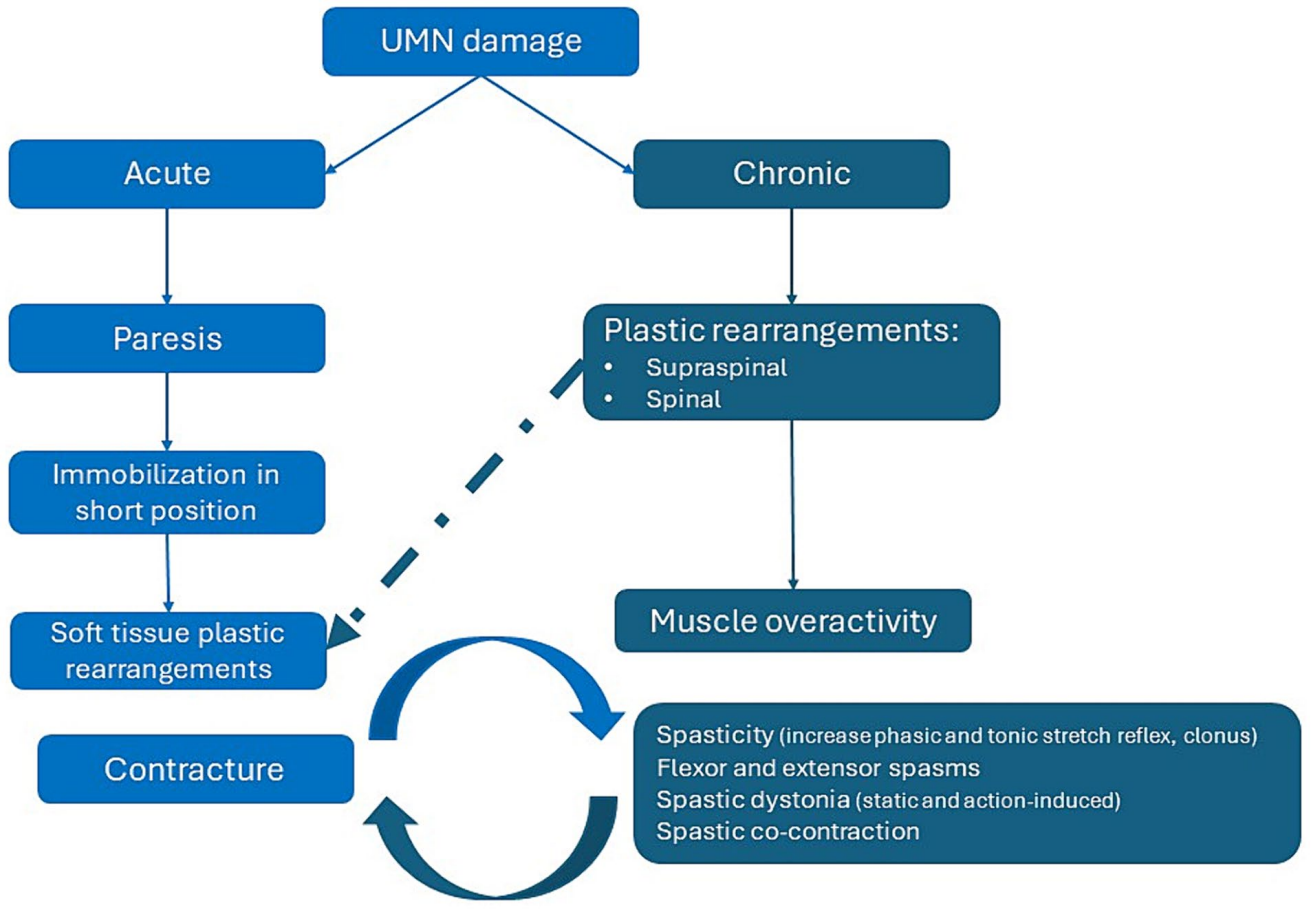
Pathophysiology of spasticity: targeting the acute and delayed phases with multimodal therapy.

There is an evolution between the acute and delayed phases of UMN damage when describing the pathophysiology of spasticity and spastic paresis, with the challenge of management addressing the ‘jigsaw’ of presentation (Figure 1). The value of including a treatment to manage one aspect of spasticity needs to be weighed against the outcomes of another, but treatment can be targeted at the CNS, peripheral and during the acute and delayed phases.

Spasticity management should be provided within the context of a comprehensive rehabilitation program. Royal College of Physician UK Guidelines (2018) state that management should be patient-specific, goal-centered, team-based and consider both generalized (i.e., posture management, physical management, dynamic movement) and focal interventions (i.e., splinting, casting), which can be considered a multimodal strategy (1). It is also important to consider the fundamental role of BoNT-A in the coordinated management of focal, multifocal, and segmental spastic paresis. Guidelines from the British Society of Rehabilitation Medicine state the importance of botulinum toxin injection as part of a rehabilitation program involving post-injection exercise, muscle stretch and/or splinting to achieve an optimal clinical effect (1).

**Figure 2:**
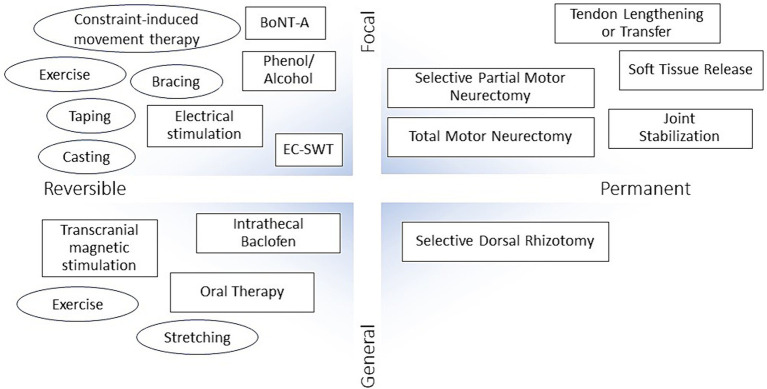
Approaches for the treatment and management of spasticity. BoNT-A, botulinum toxin type A; EC-SWT, ExtraCorporeal Shock Wave Therapy.

Treatment selection depends on understanding the role and application of each method in relation to the individual needs of the patient. In general, there a four categories of treatment approach, covering localized and reversible methods (e.g., BoNT-A, EC-SWT, and casting), focal and permanent surgical intervention, general and reversible pharmacologic therapy (e.g., intrathecal baclofen), and general and permanent selective dorsal rhizotomy (Figure 2) (10).

The concept of adjunctive therapy is to enhance the effect of an intervention by including an additional therapeutic intervention. When considering BoNT-A, examples of adjunctive therapy may involve the addition of casting, electrical stimulation, or EC-SWT to increase the therapeutic effect of BoNT-A injection; the initial treatment is considered primary, the secondary treatment is the adjunct.

Clinical experience suggests that not only does adjunctive therapy improve the uptake, effectiveness, clinical outcome of the BoNT-A injection, but such a combination approach may also reduce the dose of BoNT-A required and may, anecdotally, increase the interval to the next injection. Timing of the adjunctive therapy following BoNT-A injection is key and theoretically should be provided within 1–3 days post-BoNT-A, and up to 14 days afterwards as described in the key studies involving electrical stimulation or EC-SWT as adjunctive therapy (4, 5). However, based on the French guidelines for the use of adjunctive therapies after BoNT-A injection in spastic adults, there is variability in treatment scheduling and timing (3).

We propose that the concept of adjunctive therapy is not equivalent to multimodal therapy but is the attempt to increase the efficacy of one treatment with another, is time-based (within 2 weeks of primary therapy), and can be utilized as an aspect of multimodal therapy. Therefore, more than one adjunctive therapy can be added to enhance the efficacy of a single treatment (e.g., an attempt to enhance the effect of BoNT-A by conducting immediate electrical stimulation post-injection, followed by casting).

Multimodal therapy differs from adjunctive therapy by the addition of one or more therapeutic modalities to a current intervention to enhance the overall treatment effect, especially if the goal of the initial therapeutic interventions is not attained. Multimodal therapy is an integrated program of patient-centric, parallel or sequential treatments without a time limit. Thus, adjunctive therapy can optimize one treatment modality as part of the multimodal process. In the example described above, where attempting to enhance the effects of the BoNT-A with electrical stimulation and casting, a multimodal process may also include continuing with a self-rehabilitation program involving stretching.

Multimodal treatment and adjunctive therapy are not interchangeable terms. It is imperative to understand the differences between each method to ensure these approaches are used correctly in spasticity management to achieve treatment goals.

## Importance of goal setting when using adjunctive and multimodal therapy approaches

The importance of goal setting when planning spasticity interventions is well-established and we stressed previously how this point is crucial (11). With passive goals, treatment focuses on the positive signs of upper motor neuron syndrome (UMNS; Table 1), with more effect observed from single interventions (e.g., BoNT-A) that may not necessitate as much intensity and frequency of exercise or occupational therapy (9, 12). In relation to active goals, a ‘more is better’ approach to therapy has been proposed to help patients understand the greater amount and frequency of exercise they perform, the more likely they will achieve their goal.

**Table 1:** Signs of UMNS (39).

Positive symptoms	Negative symptoms
Spasticity	Weakness (usually extensors of the arm and flexors of the leg)
Clonus	Decreased motor control
Hyperreflexia of deep tendon reflexes	Impaired balance
Hyporeflexia of superficial reflexes	Rapid fatigue
Co-contraction	
Synkinesia	
Babinski sign (Brissaud reflex, Hoffman sign)	
Pseudo-Bulbar palsy	
Spinal shock	

Spasticity is usually accompanied by paresis, soft tissue contracture, muscle overactivity, and other signs of UMNS resulting from damage to descending motor pathways (13). The interval between injury and the appearance of spasticity may vary from days to months dependent on the level and location of the lesion (14). Increased joint stiffness in the relaxed condition can be of either neural or non-neural origin, with treatment aimed often at improving passive and active joint range of motion.

Where neural origin is suspected, reducing muscle overactivity and blocking the stretch reflex loop is often employed, while corrective casting or splinting are usually applied to alter the viscoelastic properties of muscle and connective tissues in spasticity of a non-neural origin (15).

In active and passive goals, multimodal intervention is aimed at improving the negative signs of UMNS, hence the use of more modalities may lead to a greater treatment effect (e.g., frequency of stretching, exercise).

The multimodal approach can also consider targeting the neural and non-neural peripheral elements of spasticity management, as there is a significant interplay between all these factors. While there is no current evidence of a ‘perfect recipe’ with regards to the type of multimodal intervention, a personalized ‘package’ can be based on the individual patient’s spasticity phenotype and pattern.

### Evidence for adjunctive therapy in spasticity management

A wide range of adjunctive therapies after BoNT-A injection have been proposed for managing spasticity (16–21). Although literature reviews strive to identify the most appropriate adjuvant treatment protocol after BoNT-A injection, these have yet to be defined (5).

A systematic review of randomized control studies of patients with post-stroke spasticity and multiple sclerosis identified 10 adjunctive therapies that were utilized with BoNT-A injections. Adjunctive use of electrical stimulation, modified constraint-induced movement therapy (CIMT), physiotherapy, casting, and dynamic splinting result in improved Modified Ashworth Scale (MAS) scores compared with BoNT-A injections alone, while adjunct taping, segmental muscle vibration, cyclic functional electrical stimulation, and motorized arm ergometer did not demonstrate improved outcomes vs. BoNT-A alone (4). There was evidence that casting provides improved outcomes over taping, with taping better than electrical stimulation and stretching, and EC-SWT better than electrical stimulation for outcomes including the MAS, range of motion, and gait. However, all results were based on single studies without further independent confirmation and require more extensive validation (4).

A further literature review by Picelli et al. (5) focusing on commonly used adjuvant treatments associated with BoNT-A injections for managing spasticity provided similar results (5). Adhesive taping and casting were shown to effectively improve the effect of BoNT-A in patients with upper-and lower-limb spasticity, with strong evidence that casting provided better results than taping for outcomes including spasticity, range of motion, and gait. However, there was little consensus regarding the most appropriate timing, duration, target, and material. Post-injection BoNT-A outcomes following adjunctive EC-SWT were better than with electrical stimulation for some outcome measures including spasticity and pain. Electrical stimulation of injected muscles might be useful to boost the BoNT-A effect; however, the best stimulation protocol has not been defined (5).

A literature investigation of the evidence for casting as an adjunctive therapy following BoNT-A injection for adult lower limb spasticity identified five studies across 98 participants (2 randomized controlled trials, 1 pre-post study, 1 case series, and 1 case report). Although casting protocols varied widely between studies, adjunctive casting demonstrated improvement in spasticity-related outcomes following BoNT-A injection and may result in less significant soft tissue injury compared with a stand-alone intervention. In addition, adjunctive casting was associated with improved spasticity-related outcomes compared with stretching and taping (22). Currently, there are no studies that address whether casting in addition to BoNT-A is more effective than BoNT-A alone, and consideration should be given to determine which protocols yield the best results (22).

There are no randomized control studies investigating adjunctive self-rehabilitation therapy and BoNT-A, but an international consensus statement suggests the importance of a self-rehabilitation program as an adjunctive therapy (23). Similarly, there is lack of high-quality studies for the use of robotics or transcranial magnetic stimulation as adjunctive therapy to BoNT-A injections (23).

### Obstacles to adjunctive therapy in spasticity management

Most clinicians prescribe splinting and home stretching or active exercise programs, although many patients may not be compliant or may not want such adjunctive therapies (24). A minority of practitioners, approximately a third, prescribe electrical stimulation, transcutaneous electrical nerve stimulation (TENS), casting, and EC-SWT due to lack of availability and training, potential financial costs, and perceptions of poor efficacy outcomes (24). Current international practices regarding the use of post-BoNT-A adjunctive therapies for limb spasticity highlight that active exercise or stretching programs at home, splinting, and constraint-induced movement therapy are the most used physical interventions (25). Financial and time constraints are considered barriers to implementation of adjunctive therapies, with patient preferences also potentially affecting compliance (24, 25).

A consensus paper derived from a meeting of an international group of 19 neurological rehabilitation specialists highlighted recurring practical challenges to maximizing the benefits of treating post-stroke spasticity with BoNT-A injections. Casting, splinting, and taping were considered as useful adjunctive therapies in the BoNT-A setting, but were restricted by access to casting, funding, clinician experience, and clinical time to monitor the patient post-casting. There was good evidence to consider electrical stimulation as an adjunctive therapy to BoNT-A treatment; barriers to use included lack of clinician experience, availability of device, and time constraints. EC-SWT was considered as an adjunctive therapy to BoNT-A injections, but practically difficult to implement due to costs, availability of device, and lack of clinician experience (23). The consensus group recommended that patients are trained to follow a self-rehabilitation program for spasticity and post-stroke recovery to supplement their clinician-administered physiotherapy (23).

### Evidence for multimodal therapy in spasticity management

British Society of Rehabilitation Medicine guidelines state the importance of BoNT-A as part of a rehabilitation program involving post-injection exercise, muscle stretch, and/or splinting to achieve an optimal clinical effect: these are examples of multimodal treatments.

A prospective, observational study of goal attainment in focal spasticity in adults with stroke and traumatic brain injury highlighted the utility of “a more is better approach” regarding post BoNT-A treatment modalities in a real-world practice in Australia. The authors emphasize the importance of the addition of various treatment modalities (stretching, electrical stimulation, task specific practice, splinting, serial casting, robotic) with BoNT-A injections to increase the probability of attaining active goals. This study of 38 patients supports an important consideration for future studies, highlighting that the addition of numerous treatment modalities after BoNT-A treatment, rather than the type of treatment modality, is vital to attaining active goals (9).

In a randomized, controlled, double-blind trial, Leung et al. (8) demonstrated a 26-degree improvement in passive ankle dorsiflexion range at the Week 2 and Week 8 assessments with a combination of BoNT-A injection plus serial casting and motor training followed by splinting; the control group underwent a 6-week wait followed by the same interventions as the treatment group. The outcomes from this study of patients with severe acquired brain injury (n = 10, 13 ankles) show the importance of using combination therapy or multimodal therapy to increase treatment outcomes in contracture management (8).

A prospective study of 10 patients with spasticity secondary to acquired brain injury examined BoNT-A injections plus casting and a home exercise stretching program as an example of adjunctive therapy to enhance the toxin effect and use of home exercise as a multimodal treatment approach, with goals of improving elbow spasticity, improving gait with upper extremity spasticity treatment alone, and increasing the BoNT-A injection interval. Patients received BoNT-A injections under ultrasound guidance and, at 2 weeks post-injection, an elbow stretching cast was applied for 1 week (26). Outcomes show that the multimodal combination decreased elbow spasticity by 80% and improved hemiparetic gait parameters without treatment of the lower limbs. In addition, there were trends for a longer duration between BoNT-A treatment intervals (26).

A recent study by Suputtitada et al. (27) revealed that there was Grade A evidence for multimodal therapy in the treatment of post-stroke spasticity and that stretching exercises, static stretching with positional orthosis, transcutaneous electrical nerve stimulation, extracorporeal shock wave therapy, peripheral magnetic stimulation, non-invasive brain stimulation, botulinum toxin A injection, dry needling, intrathecal baclofen, whole body vibration, and localized muscle vibration were supported by Grade A evidence as effective for improving functional recovery and quality of life in post-stroke spasticity (27). However, the focus was on high-quality medical, rehabilitation, and surgical evidence only, identifying 32 treatments. Interventions with low-grade outcomes were not considered except for static stretching with positional orthosis in a neutral or extended wrist position, which were considered superior to no therapy in reducing wrist flexor spasticity in chronic stroke patients. Due to factors influencing low-quality studies, such as low sample sizes and high levels of heterogeneity, caution is warranted when drawing conclusions (27). Suputtitada et al. highlighted that multimodal therapies with high-quality evidence for post-stroke spasticity may offer superior outcomes compared with oral medications, which have the potential to impede functional recovery (27).

**Figure 3:**
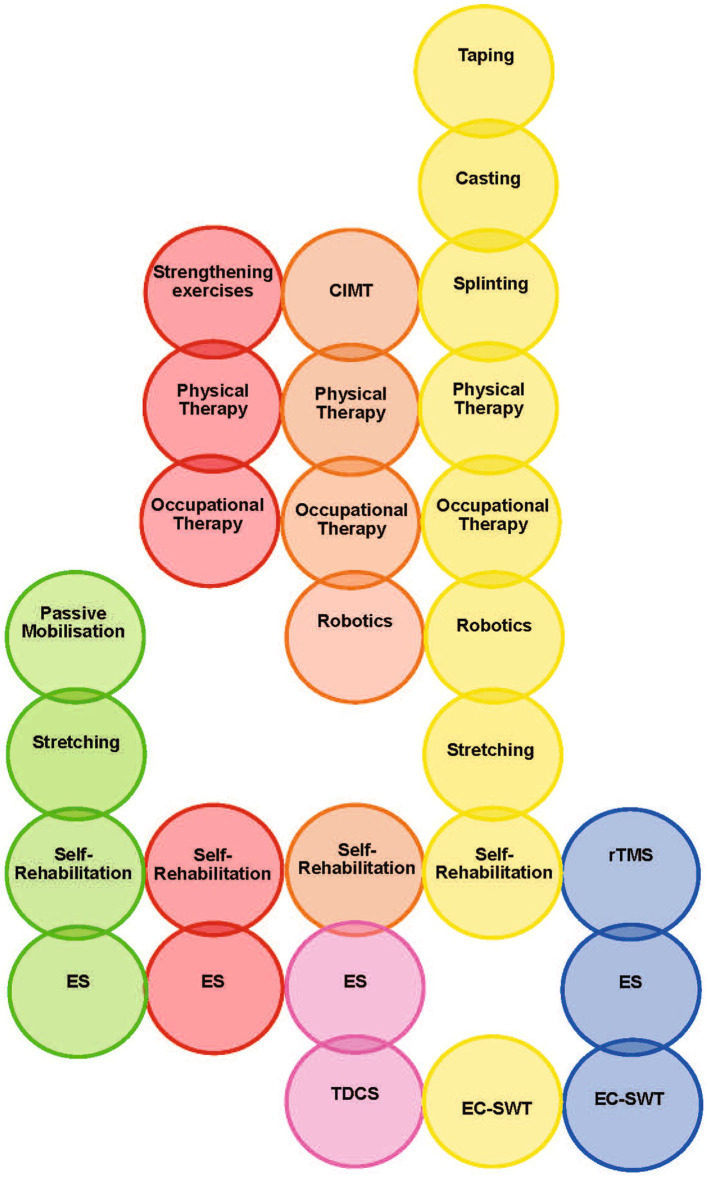
Proposed rational for using multimodal non-pharmacologic treatments for spastic movement disorder (21, 23). Red, Increase Antagonists Strength; Yellow, Improve Rehological Properties of Spastic Muscles/Improving ROM; Orange, Improve Motor Control; Green, Improve Pain Control; Blue, Tonus Reduction of Spastic Muscles; Pink, Facilitation of Spinal Motoneuron Excitability ES, Electrical Stimulation; EC-SWT, ExtraCorporeal Shock Wave Therapy; CIMT, Constraint-Induced Movement Therapy; rTMS, repetitive Transcranial Magnetic Stimulation; TDCS, Transcranial Direct Current Stimulation; ROM, range of motion.

Conservative therapeutic modalities should be considered for the neural and non-neural mechanism of action, with the most effective modality potentially acting on both; more modalities will be required to address active rather than passive goals (28). Modalities that include psychological biofeedback may also be part of the non-motor element of spasticity management as part of the multimodal approach. Although evidence levels supporting combination therapies are even lower than those of individual treatment approaches, and outcomes are dependent on individual patient goals, possible multimodal treatments following BoNT-A for Spastic Movement Disorder are proposed in Figure 3 (21, 23).

**Figure 4:**
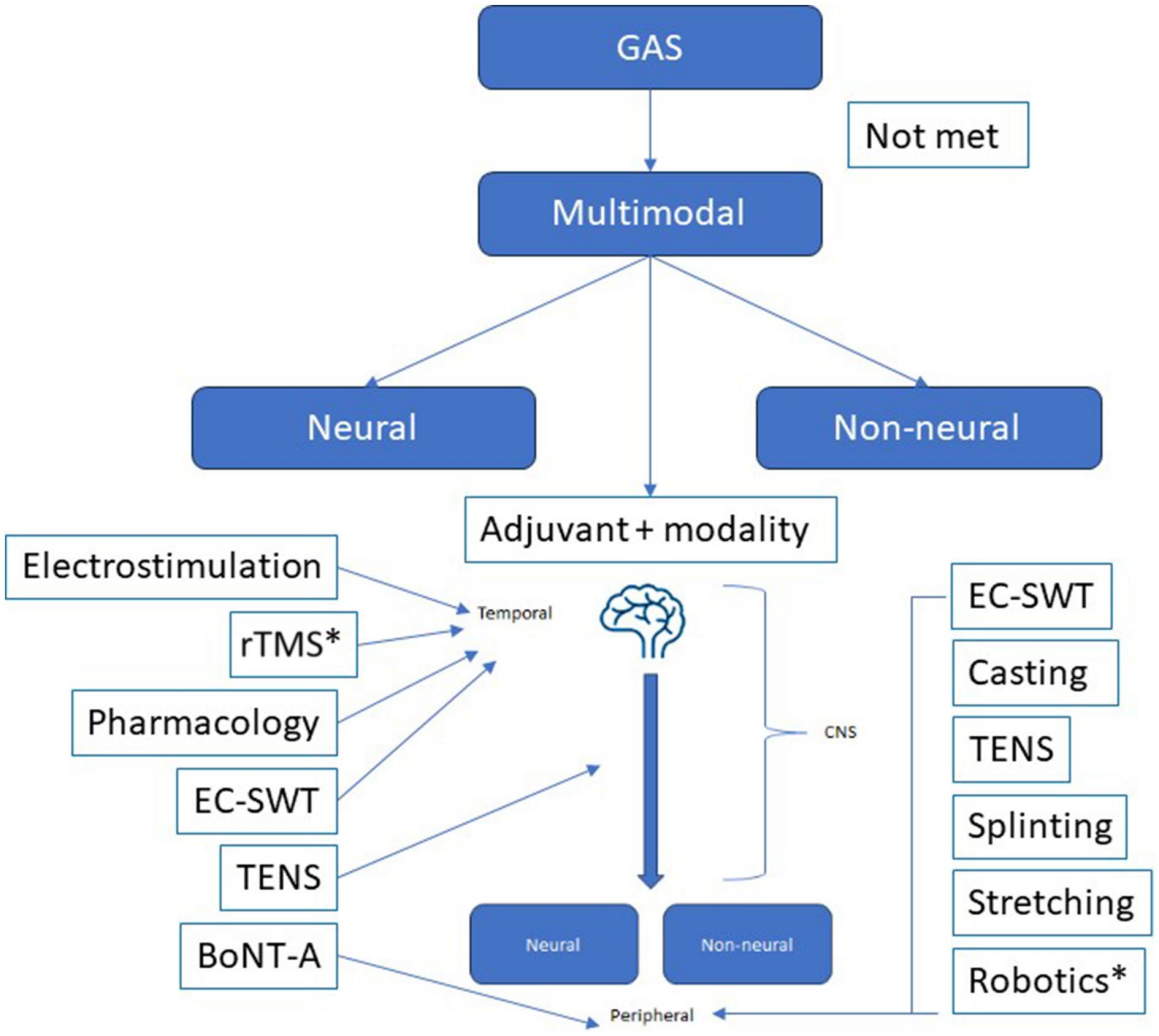
Multimodal therapy can target both neural and non-neural pathways, as well as help with soft tissue rearrangements and decrease muscle overactivity. BoNT-A can be part of the multimodal therapy and as an adjunct to other treatments (such as casting, electrical stimulation, splinting, physiotherapy), or treatments such as casting, electrical stimulation, splinting, physiotherapy can be an adjunct to BoNT-A injections. *Consensus on utility not obtained from the TOXNET group. GAS, goal attainment scale; EC-SWT, ExtraCorporeal Shock Wave Therapy; TENS, transcutaneous electrical nerve stimulation; rTMS, repetitive transcranial magnetic stimulation.

This discussion paper has focused on conservative modalities for adjunctive and multimodal strategies with BoNT-A. A multimodal approach can also include the use of pharmacological/pharmaceutical and surgical interventions. Surgery, such as tendon transfers or tenotomy, may be considered following worsening or soft tissue retraction and fixed contracture. Multimodal therapy can target both neural and non-neural pathways, as well as help with soft tissue rearrangements and decrease muscle overactivity (Figure 4). At present, there is no consensus on the minimum or maximum number of multimodal therapies required to enhance spasticity treatment outcomes.

While there is a growing trend in clinical practice towards adopting a multimodal approach, several obstacles hinder the wider utilization of such therapies. Recent consensus-led work by Patel et al. (2023) suggests that the key barriers to spasticity care relate to access of care, caregiver and community awareness of spasticity, and clinician education (29). In addition, insufficient training and clinical protocols, as well as limitations in healthcare accessibility, and patient affordability have been identified as hurdles to accessing post-stroke care (1, 13, 30, 31).

During the first 12 months after the stroke, costs for patients who develop spasticity can be up to four times as high as those for patients who do not (30, 31). Direct costs in spasticity management include diagnostic procedures, specialist referrals, medications, non-pharmacological therapies, hospitalizations, medical devices, and nursing home care, while indirect costs include loss of productivity due to PSS for patients engaged in gainful employment (30, 32).

Awareness of available treatment modalities is critical for care access and referral. While it is generally accepted that centers will not have access to every treatment, it is important to understand the spectrum of options available to specialist spasticity services for an effective referral process (1, 13).

### Emerging therapies in spasticity management

Several emerging therapies and technologies have the potential to integrate within the current framework of multimodal and adjunctive therapies. Currently, such interventions have low-grade outcome evidence and cannot be recommended directly as post-stroke spasticity therapy following BoNT-A injections (21).

Two narrative reviews of repetitive transcranial magnetic stimulation (rTMS) and transcranial direct current stimulation (tDCS) reported that low-frequency rTMS over unaffected hemispheres may be effective in reducing spasticity when applied alone or with conventional therapies, but there is a need for uniform, large, multicenter trials (33, 34).

A systematic review by Hofmeijer et al. (35) of 57 articles (*N* = 2,595) suggested that rTMS holds the potential to benefit a range of motor and cognitive outcomes after stroke, but the current evidence is challenged by unexplained heterogeneity across many small-sampled trials (35). Current mechanism research is preliminary, and a more thorough understanding of underlying rTMS-mediated CNS regulation may help in the better application of rTMS to clinical treatment (36).

Non-invasive neuromodulation with tDCS has shown promise in improving rates of motor recovery following stroke, with numerous studies suggesting efficacy, particularly as an adjunct to rehabilitation. A major hurdle for tDCS is the high degree of intra-and inter-subject variability and there remains widespread disagreement regarding the source of inconsistent outcomes resulting from tDCS administration (37).

Little robust evidence exists concerning the use of robotics in spasticity. A review of electro-mechanical and robot-assisted arm training concluded that the quality of evidence is low, and studies show too much heterogeneity in intensity, duration, amount of training, type of treatment, and participant characteristics to make meaningful conclusions (38).

## Discussion

Currently, there is a general clinical trend towards including casting, electrical stimulation, or EC-SWT as adjuncts to BoNT-A injections, or as part of an overall multimodal approach (13, 22–24). However, barriers to wider use of such therapies include a general lack of training and clinical protocols, as well as both healthcare and patient financial limitations (5, 18, 23–25).

In summary, multimodal spasticity management employs various pharmacologic and non-pharmacologic strategies concurrently or sequentially to address the variety of impairments associated with spasticity (e.g., neural and non-neural components). These combined therapies are identified *a priori* following goal-setting and clinical evaluation. Adjunctive therapies, on the other hand, describe pharmacologic and non-pharmacologic strategies that are individually chosen to optimize the efficacy of a particular intervention, and are usually administered after the main intervention has been delivered. Individual adjunctive therapies may be part of a multimodality treatment approach if they are regarded with equal importance as other concurrent interventions, rather than viewed as supportive measures.

## Data Availability

The original contributions presented in the study are included in the article/supplementary material, further inquiries can be directed to the corresponding author.
